# Eptifibatide and Cirrhosis: Rethinking GPIIb-IIIa Inhibitors for Acute Coronary Syndrome in the Setting of Liver Dysfunction

**DOI:** 10.14740/cr357w

**Published:** 2014-12-04

**Authors:** Michael Weinreich, Dexter Mendoza, Thomas Pettei, Evelina Grayver

**Affiliations:** a Department of Medicine, North Shore Long Island Jewish Health System/Hofstra University School of Medicine, Manhasset, NY, USA; b Department of Cardiology, North Shore Long Island Jewish Health System/Hofstra University School of Medicine, Manhasset, NY, USA

**Keywords:** GPIIb/IIIa inhibitors, Integrilin, Acute coronary syndrome, Thrombocytopenia, Cirrhosis, Chronic liver disease

## Abstract

Glycoprotein IIb/IIIa (GPIIb/IIIa) inhibitors, such as eptifibatide, are routinely used in the setting of acute coronary syndrome (ACS) prior to or during percutaneous coronary intervention (PCI). While numerous studies have demonstrated improved clinical outcomes with eptifibatide use, adverse effects including thrombocytopenia have also been noted. For this reason, patients with baseline thrombocytopenia or liver disease should be cautiously evaluated prior to drug administration. Here we report a case of acute profound and prolonged eptifibatide-induced thrombocytopenia in a patient with cirrhotic liver dysfunction. We propose and discuss the need for a risk stratification tool to be established for identifying which patients with ACS in the setting of chronic liver disease receive GPIIb/IIIa inhibitors.

## Introduction

The standard management of acute coronary syndrome (ACS) has traditionally been a constellation of percutaneous coronary intervention (PCI), aspirin, thienopyridines, and heparin. Clinical trials over the last 17 years, however, have shown that the addition of glycoprotein IIb/IIIa (GPIIb/IIIa) platelet inhibitors improves cardiovascular outcomes [1-4]. Despite these benefits, many case reports have demonstrated clinically significant side effects of GPIIb/IIIa inhibitor use, the most notable of which is thrombocytopenia [5-7]. Two recent case reports have further demonstrated thrombocytopenia accompanied with disseminated intravascular coagulation (DIC) and/or thrombosis in patients administered eptifibatide [8, 9]. Others have reported acute profound thrombocytopenia [10]. As a result, it is vital to identify which patients are at highest risk for the development of thrombocytopenia and avoid use of GPIIb/IIIa inhibitors in these groups. While anti-platelet agents are routinely used in cirrhotic patients undergoing PCI, the data on complications and bleeding risks are limited. Further research needs to be performed to delineate other groups, in addition to liver disease patients, who may be at risk for acute profound and prolonged thrombocytopenia in GPIIb/IIIa inhibitor use.

## Case Report

A 62-year-old female, with history of obesity, type II diabetes mellitus, and hypertension presented to an outside hospital emergency department for chest pain. She described the pain as 10 out of 10 in severity, burning in nature, with radiation to her back and left shoulder. She had similar pain intermittently for the 3 months prior to presentation, previously alleviated by over-the-counter antacids, but was not helped during this episode. An esophagogastroduodenoscopy (EGD) a few months prior revealed moderate gastritis and an angiogram a year prior showed minor luminal irregularities without any flow limiting lesions. Her other medical history included stage IV non-alcoholic hepatic cirrhosis (confirmed with ultrasound imaging) and a history of laparoscopic gastric banding. Her home medications included metoprolol, metformin, omeprazole, and calcium carbonate. She reported that her father had a myocardial infarction in his sixties. She had a 46-pack-year smoking history.

At the outside facility, the patient was found to have positive cardiac biomarkers and ST changes on the anterior leads. She was given aspirin 325 mg, clopidogrel 300 mg, and started on a heparin infusion. She was then transferred to our facility for further management and possible PCI.

Upon arrival to our facility, her echocardiogram showed Q waves and ST changes in the anterior leads. Cardiac markers included creatine kinase (CK) of 203 ng/mL, CK myoglobin (CK Mb) of 2 ng/mL, and troponin T of 0.07 ng/mL. A complete blood count (CBC) obtained at the time of presentation revealed a white blood cell (WBC) count of 8.9 × 10^3^/mm^3^, hemoglobin of 13.8 g/dL, and mild thrombocytopenia with platelet count of 116 × 10^3^/mm^3^. A comprehensive metabolic panel (CMP) drawn at the same time included serum creatinine of 0.90 mg/dL, aspartame aminotransferase (AST) of 32 U/L, alanine aminotransferase of 35 U/L, and alkaline phosphatase of 85 U/L. The remainder of her liver function tests, including albumin and bilirubin levels and coagulation panel, were all within normal limits. Review of records revealed that the CBC and CMP were similar with previous prior testing. Of note, her platelet count 7 months earlier was 102 × 10^3^/mm^3^ and records from 4 years earlier showed it was 121 × 10^3^/mm^3^. She was continued on a heparin drip and eptifibatide infusion was initiated in preparation for cardiac catheterization with possible intervention. She was also continued on aspirin, clopidogrel, and metoprolol. The patient received intravenous nitroglycerin, which improved her pain.

Approximately 4 h after initiation of eptifibatide infusion, the patient had a precipitous reduction in platelets, dropping by more than 95% from her baseline to 3 × 10^3^/mm^3^. The rest of her CBC, including hemoglobin and WBC, remained within normal limits. Eptifibatide, heparin, aspirin, and clopidogrel were promptly discontinued and serial monitoring of her platelet count was initiated. ELISA testing for antibodies against heparin-PF4 and serotonin releasing assay were negative, ruling out heparin-induced thrombocytopenia. In addition, her coagulation panel remained within normal limits. Based on the time course of the thrombocytopenia and her Naranjo algorithm score, it was believed that the reduction in platelet count was secondary to eptifibatide use [11].

After discontinuation of eptifibatide, the platelet count rose gradually, reaching 7 × 10^3^/mm^3^ 8 h after the discontinuation of the drug and 19 × 10^3^/mm^3^ approximately 24 h later. Given the expected short half-life of eptifibatide (2.5 h), the patient did not receive a platelet transfusion. Her platelet count continued to rise without any transfusion, reaching 27 × 10^3^/mm^3^, 47 × 10^3^/mm^3^, and 71 × 10^3^/mm^3^ at approximately 48, 72, and 96 h after discontinuation of eptifibatide, respectively. At the day of discharge, approximately 6 days after discontinuation of eptifibatide, her platelet count had reached 147 × 10^3^/mm^3^. She did not exhibit any signs of active bleeding or bruising at any point during her hospital stay.

Due to the profound thrombocytopenia, cardiac catheterization was delayed. A viability study was conducted, which showed severe defects in the apical and distal anterior wall with nonviable myocardium. On day 7 of hospitalization, the patient underwent an angiogram to characterize the extent of coronary disease supplying her viable myocardium. The angiogram revealed 100% occlusion of the left anterior descending and minor disease of the right coronary and circumflex arteries. No percutaneous intervention was undertaken given the results of viability study. The patient was subsequently discharged on aspirin, atorvastatin, metoprolol, and lisinopril.

## Discussion

Eptifibatide is a synthetic cyclic heptapeptide which functions via competitive inhibition of the platelet GPIIb/IIIa receptor, thereby preventing fibrinogen-platelet aggregation, a precipitant of thrombus formation [9]. Thrombocytopenia is a well-documented adverse effect associated with the use of eptifibatide in the setting of both ACS and as prophylaxis against stent restenosis in patients with malignancy [5-9]. The PURSUIT Trial, the landmark study which hailed the use of GPIIb/IIIa inhibitors, itself reported an increased incidence of platelet counts below 20 × 10^3^/mm^3^ in those receiving the study drug as compared with placebo (0.2% vs. < 0.1%; relative risk: 5.0; 95% confidence interval: 1.3 - 32.42) [2].

Many case reports have remarked on the rapid and precipitous drop in platelet count associated with eptifibatide use, prompting the recommendation for monitoring of serum levels within 2 - 6 h of initiation of GPIIb/IIIa inhibitor administration [12]. The biochemical mechanism of this finding remains unclear. The time course suggests both a non-immune and immune mechanism is likely. Bougie et al (2002) report a series of patients with eptifibatide-induced thrombocytopenia as well as a high titer of IgG antibody that reacted with the GPIIb/IIIa complex only in the presence of the drug used in treatment [13]. Their results showed that the adverse effect was likely secondary to drug-dependent antibodies that may be either naturally occurring or induced by previous interactions. Other studies have correlated previous exposure to eptifibatide and findings of thrombocytopenia [14, 15].

In our case, a patient with known cirrhotic liver dysfunction experienced profound (a decrease in platelet count to < 20 × 10^3^/mm^3^ within 24 h) and prolonged (> 4 days) thrombocytopenia just 4 h after a single dose of eptifibatide [10]. Regardless of the biochemical pathway, it is significant in that the clinical event delayed time to percutaneous intervention, as the bleeding risk was too great. This delay in treatment arguably altered the course of reperfusion and outcome of the patient’s viable myocardium.

In review of our patient management, integrilin (Merck & Co., Inc., Whitehouse Station, NJ) was likely not the optimal therapeutic choice for our patient, as she had both baseline thrombocytopenia and liver dysfunction. Thrombocytopenia is a well-established consequence of liver dysfunction. Although the association is clear, the pathophysiologic mechanism for this finding remains under study. The classic process is believed to be secondary to splenic sequestration associated with portal hypertension. More recent investigations have suggested the culprit to be decreased production of thrombopoietin (TPO) by injured hepatocytes [16]. Those with alcoholic cirrhosis may also suffer from marrow suppression or folic acid deficiency. Those with cirrhotic liver failure usually maintain platelet levels above 50 × 10^3^/mm^3^.

Consequently, patients such as the one described in this report, with baseline thrombocytopenia and liver dysfunction, should be considered at high risk when administered anti-platelet agents, especially GPIIb/IIIa inhibitors. Eptifibatide has a plasma elimination half-life of approximately 2.5 h, and in healthy subjects, renal clearance accounts for approximately 50% of total body clearance. Its effects on liver and liver disease are largely unknown. Curiously, the Canadian package insert for integrilin lists clinically significant liver disease as an absolute contraindication to use of the drug [17]. In the United States, the package insert does not list this recommendation and states only that “no studies have been conducted in patients with hepatic impairment” [18]. Despite these inconsistencies, the risk to benefit ratio for the use of anti-platelet agents in liver disease is a topic of emerging discussion within hepatologic societies.

In addition to the traditional view of an increased bleeding tendency and hypocoagulability among chronic liver disease patients, it is now believed that these patients suffer from a spectrum of coagulability disorders, including a propensity to form venous and arterial thromboses. Risk stratifying these patients and selecting an anti-platelet agent in the setting of ACS remains an area of uncertainty among clinical physicians. Guidelines for the pharmacologic treatment or prevention of thrombotic events in patients with liver disease are yet to be developed [19]. Lisman et al (2013) provide a comprehensive review of the anti-platelet agents and their risks/benefits in liver disease, but do not comment on GPIIb/IIIa inhibitors [19]. The authors do, however, recommend the use of primary and secondary prevention anti-platelet agents in high risk liver disease patients with arterial thrombotic events, including coronary thrombosis.

Patients with liver cirrhosis placed on the transplant list are routinely screened for coronary artery disease (CAD) prior to surgical intervention. Often, these patients undergo cardiac catheterization and PCI with either bare metal or drug-eluting stents requiring long-term anti-platelet agents. Russo et al (2012) retrospectively evaluated a group of 423 cirrhotic patients undergoing transplant evaluation for CAD [20]. Of those who underwent PCI and were subsequently placed on anti-platelet therapy, there was no significant increased risk of bleeding as compared to a control group (12.5% vs. 6.3%; P = 0.86) [20]. However, larger studies are necessary to confirm these findings given the trend toward an increased bleeding risk. As the incidence of non-alcoholic fatty liver disease (NAFLD), non-alcoholic steatohepatitis (NASH), alcoholic cirrhosis, and viral hepatitis increases concurrently with the incidence of ACS, a continual focus on addressing the side effects, bleeding profiles, and indications for these drugs is warranted.

### Conclusion

The addition of GPIIb/IIIa platelet inhibitors to the traditional therapy of PCI, aspirin, thienopyridines, and heparin has led to improvement in cardiovascular outcomes in patients experiencing ACS. However, GPIIb/IIIa inhibitor use should be carefully considered in patients with known liver dysfunction. As the “obesity epidemic” expands within the United States and around the world, so will the incidences of NAFLD, NASH, and ACS. We propose that a risk stratification tool be developed for identifying which patients with ACS in the setting of chronic liver disease should receive GPIIb/IIIa inhibitors. Further research is needed to delineate other groups, aside from liver disease, which may be at risk for acute profound and prolonged thrombocytopenia in GPIIb/IIIa inhibitor use.

### Learning objectives

GPIIb/IIIa inhibitors, such as eptifibatide, are routinely used in the setting of ACS prior to or during PCI. However, use of this class of medication has been associated with thrombocytopenia. Administration of GPIIb/IIIa inhibitors in the setting of liver dysfunction has not been adequately studied. Further research is needed to risk stratify patients with liver disease at risk for thrombocytopenia.
